# Sentence vs. Word Perception by Young Healthy Females: Toward a Better Understanding of Emotion in Spoken Language

**DOI:** 10.3389/fgwh.2022.829114

**Published:** 2022-05-25

**Authors:** Rachel-Tzofia Sinvani, Shimon Sapir

**Affiliations:** ^1^School of Occupational Therapy, Faculty of Medicine, The Hebrew University of Jerusalem, Jerusalem, Israel; ^2^Department of Communication Sciences and Disorders, Faculty of Social Welfare and Health Sciences, University of Haifa, Haifa, Israel

**Keywords:** speech recognition, emotion, utterances, perception, *word vs. sentence*, female, gender, Hebrew

## Abstract

Expression and perception of emotions by voice are fundamental for basic mental health stability. Since different languages interpret results differently, studies should be guided by the relationship between speech complexity and the emotional perception. The aim of our study was therefore to analyze the efficiency of speech stimuli, *word* vs. *sentence*, as it relates to the accuracy of four different categories of emotions: **anger**, **sadness**, **happiness**, and **neutrality**. To this end, a total of 2,235 audio clips were presented to 49 females, native Hebrew speakers, aged 20–30 years (M = 23.7; SD = 2.13). Participants were asked to judge audio utterances according to one of four emotional categories: anger, sadness, happiness, and neutrality. Simulated voice samples were consisting of words and meaningful sentences, provided by 15 healthy young females Hebrew native speakers. Generally, *word* vs. *sentence* was not originally accepted as a means of emotional recognition of voice; However, introducing a variety of speech utterances revealed a different perception. Thus, the emotional conveyance provided new, even higher precision to our findings: **Anger** emotions produced a higher impact to the single *word* (χ^2^ = 10.21, *p* < 0.01) as opposed to the *sentence*, while **sadness** was identified more accurately with a sentence (χ^2^ = 3.83, *p* = 0.05). Our findings resulted in a better understanding of how speech types can interpret perception, as a part of mental health.

## Introduction

Humans use a range of cues to communicate emotions, including facial expressions, posture, and vocalizations (1). Although encoding, decoding, and interpreting emotions are, by default, a multisensory integration process, one channel is more than sufficient at deciphering a person's emotional state well above chance (2, 3). However, the voice is a highly complex tool of communication. Research showed that gender played a key role in auditory, visual, and audio-visual modalities (4, 5), with females outperforming males in all three conditions of stimulus presentation (6). More specifically, studies within different cultures, demonstrated a significant female advantage in emotional recognition which was however restricted to vocal emotions [e.g., (7–10)]. Moreover, gender had been revealed as predominant in expression of emotions by voice (5, 11). As shown previously, results indicate a higher accuracy level when spoken by female compared to male actors (9, 12, 13).

Regarding the vocal stimuli, the different types of vocal cues that are overlayed onto words, pseudo-words, sentences, pseudo-sentences, affected bursts etc. capture the pure effects of emotional prosody. Separate from the lexical-semantic cues, sense word and other speech stimuli have offered communicational value [(14); A detailed review of emotional speech databases can be found in Gangamohan et al. (15)], and are an ideal tool when investigating the expression of emotions (16, 17). However, in order to fully understand the emotional information, we tend to combine the information of verbal and non-verbal vocalizations into multiple communication modules (18, 19). In other words, verbal cues are the “what is being said” as opposed to non-verbal cues, which translates to “how one says it.” Moreover, comparing voice patterns, speech is the most natural way of expressing oneself as human. Cross culturally, *word*s vs. *sentence*s are used to express contextual as well as non-verbal information (20–23). Together they can paint a vivid picture for communication. Such vocal communication influences the perception of the listener to the speaker (24). However, deficits in emotional recognition related to failures in semantic or prosodic extraction, have been associated with depression (25), aging (26), hearing impairment (27, 28) as well as other neurodegenerative diseases: i.e., Parkinsons (29), Autism and other neurodevelopmental disorders (30). While affect-bursts have been studied cross-culturally, speech stimuli remain restricted to specific language currently under study. Therefore, recent studies involved one language due to the rationale that each language has it's own cultural characteristics which should be studied independently (8, 12, 31).

Several research fall into the scope of emotional speech and are characterized differently. In order to overcome the challenge of the various methodological issues, in the current study, we adopted previous recommendations, accordingly, which targeted the more unified sample making it theoretically possible for more definitive findings (32). In the current study, our aim was therefore to test the advantage of speech stimulus types (word vs. sentence) in Hebrew language, based on the female's advantaged perspective. Thus, we sampled only healthy young females, of which, all were Hebrew native speakers. Our hypothesis submitted that as long as females were more sensitive to extracting emotional cues, a potential difference between both type of speech stimuli would be revealed. we investigated certain emotions, verbalized differently, but communicated the same affected states. As far as we know, this is the first study to compare the efficacy of different verbal categories producing the same emotional recordings. The current investigation of the neutral statement [/Ma/Be/e/met/A/ni/lo/ /Ma/a/mi/na/] (“Oh really? I can't believe it!”) on varying degrees of affected speech, illustrate our point about how prosodic cues, rather than semantics, impact the results. Thus, the premise of this study was that sentence would enable emotional expression more clearly than the use of word. Prior to our investigation, [/Anna/] was predominantly used to imply neutrality, thereby highlighting varying degrees of emotions, not corrupted by emotional effects (29). Additionally, we predicted that by using acted speech utterances, these enhanced methods would differentiate between perception of emotion through replication or enhancement.

## Methods

### Stimuli

A total of 2235 audio clips of acted anger, sad, happy and neutral voices expressions were used. Two types of speech stimuli were illustrated by [/Ma/Be/e/met/A/ni/lo/ /Ma/a/mi/na/] **(“Oh really? I can't believe it!”)** and [/Anna/] **(subject's name)** which produced different contrived emotional expressions. Building a corpus of affected speech requires us to include a perception study, considering several important methodological issues. To this end, a pilot study confirmed that the collected audio clips were sufficiently recognizable (80% mean recognition rate). Noting the variability of the findings in the affected speech, multiple speaker recordings were designed. Rather than utilizing exaggerated emotional patterns we decided to implement the process using nonprofessional actors for these recordings. Similar audio clips following these procedures were recently implemented (31). The speech recordings were obtained in a sound-treated booth using a head mounted condenser microphone (AKG C410) positioned 10 cm and 45°-50° from the left oral angle. The signal was preamplifier, low pass-filtered at 9.8 kHz and digitized to computer hard disk at a sampling rate of 22 kHz using the PRAAT software (33). The full set of audio clips is available upon request from the authors.

### Participants

The study used a cross-sectional design and a convenience sample of undergraduate students, Hebrew speakers at University of Haifa, Israel. Exclusion criteria were extensive cognitive disabilities such as intellectual disability, psychiatric disorder or total hearing or visual loss. The recording stage involved 15 speakers. All of them were non-professional actors females, 22–26 years old (M = 24.9, SD = 2.4). For the perception test, 49 non-actors females between 20–30 years old (M = 23.7, SD = 2.13) judged the collected audio clips. Sample size was determined based on effect sizes in previous studies with emotional stimuli [e.g., (24, 34)], and was estimated to be of sufficient power for detecting a medium to large effect size. All experiments were approved by the IRB ethics committees at the University of Haifa, Israel. All subjects receiving course credit for their participation. Distinguishing between professional vs. non-professional subjects, we specified amateur participants rather than trained actors. This allows us to ensure our study is focused on female's production and perception of emotion, rather than potential sensitivity as a result of professional experience.

### Procedure

A simulated emotional paradigm was used for this recording process. The same utterance was enacted each time, to produce certain affected states [/Ma/Be/e/met/A/ni/lo/ /Ma/a/mi/na/] meaning (“oh really, I can't believe it!”). Instructed to imagine a contrived situation, these actors replicated the desired conditions shown on the computer screen. Using the idea of semantic utterance, they would produce different emotional states. Four affected states were recorded according to this methodology: **anger, happiness, sadness**, and **neutrality**. These all are often referred to as basic emotions (35). Control of such readings were organized into five groups. For two of the groups, the first type of speech was the *sentence*, followed by the *word*. For the other three groups, the first type of speech was the *word* followed by the *sentence*. Randomized procedures were executed by each group as to not be diminished by emotion.

All voice clips were judged by a total of 49 listener, as participants were asked to judge the corpus of each emotional expressions, according to one of four categories: **anger, sadness, happiness, neutrality**.

All the stimuli were presented randomly using standard headphones (Sony MDR-7506). The lead time of the total process was approximately 10 min, subdividing each audio clip into separate frames. Here we used a fixed-choice response format which follows the listeners, allowing them to listen to the stimuli once, and be directed to choose one of four affected states listed on the computer screen. By using PRAAT (33), we eliminated on-average silent recordings compared to other audio clips collected. Additionally, we had to exclude from the final sample those recordings that do not contain all the required recordings (missing data). Thus, 117 audio clips were excluded resulting in 2,235 being evaluated at the perception stage.

### Empirical Analysis

We transformed the response variable into a binary indicator of whether an affective state encoded in the vocal stimuli is successfully identified or not (0 = not identified, 1 = successfully identified). Perception test results were presented through a matrix in **Table 2**, in which the encoded emotions are plotted against the decoded emotions. This has allowed us to study the percentages of correct responses over the patterns of confusion.

Chi-square tests for independence with Yates corrections were conducted for investigating theoretical differences between *word* vs. *sentence* in total numbers of judgments, comprising each emotional category. In further steps, Chi-square tests for independence were conducted for investigating differences between the various emotional categories within the same speech type. In all Chi-square tests, the correct number of judgments and the incorrect number of judgments were used in the calculations to compare accuracy percentages.

## Results

**Figure 1:**
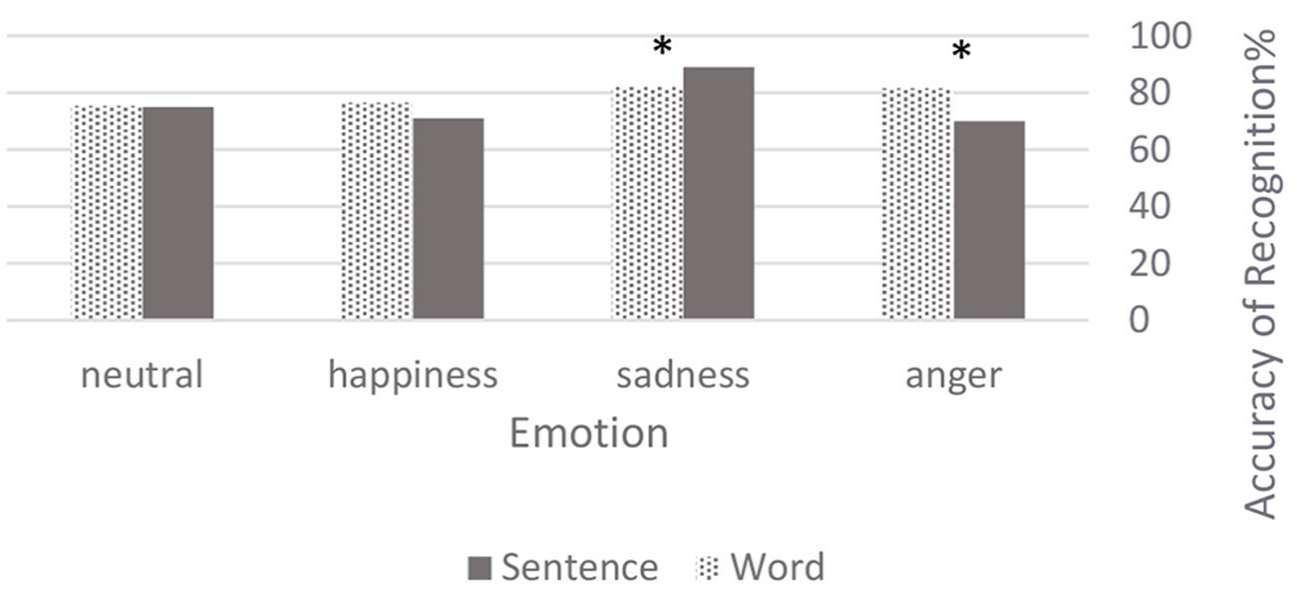
Accuracy level of emotional perceptions. Values are shown in percentages. The starred tables show significant differences between types of speech by emotion. *p < 0.05.

A total of 2,235 voice samples were judged by 49 subjects (Table 1). Results of the identification test, form a set of categorical data, which were reported in confusion matrices: Table 2 reflects the accuracy level of all emotions in the *word* vs. *sentence* expressions. Within the overall accuracy level, these emotions exceeded the chance level (75.8, 85.7, 73.7, and 75.2% regarding **anger**, **sadness**, **happiness**, and **neutrality** respectively). In conclusion, based on the Chi-square tests, our findings were inconclusive as to which input was better. However, we did find that **anger** was more definitive with *word* (χ^2^ = 10.2182, *p* < 0.05), whereas **sadness** was perceived as better in *sentence* (χ^2^ = 3.8319, *p* < 0.05). Figure 1 expressed differences between emotional perceptions through *sentence* vs. *word*.

**Table 1:** Parameters of speech analysis conducted.

	**Anger**	**Sadness**	**Happiness**	**Neutrality**	Total*
Sentence	271	272	272	267	1082
Word	289	290	285	289	1153
Total	560	562	557	556	2235

*Total numbers of recorded audio clips*.

**Table 2:** Accuracy level for differing emotions within *word* vs. *sentence* model.

**Targeted**	**Anger**	**Sadness**	**Happiness**	**Neutrality**
**emotions:**	**Word/sentence**	**Word/sentence**	**Word/sentence**	**Word/sentence**
Anger	**81.7/69.7**	2/2.9	11.2/13.6	6.2/10.5
Sadness	4.1/2.9	**82.4/88.6**	4.2/1.8	10.7/9
Happiness	6.2/14	3.4/3.7	**76.5/70.9**	7.6/5.6
Neutrality	7.9/13.3	12/4.8	8/13.6	**75.4/74.9**

*Bold number presents actual reliable statistics; Un bolded represent errors in the perceptual test.*

*The values are shown as percentages*.

In analyzing emotions within *sentence* recordings, **sadness** was relayed more effectively, as compared to other emotions (χ^2^ = 33.5319, *p* < 0.05). Conversely, in the *word* recordings there were no significant disparities between the different emotions (χ^2^ = 6.5714, *p* = 0.87).

## Discussion

In the current study, we defined accuracy of emotional perception universal, including Hebrew native speakers using *word* and *sentence* communications. Additionally, we pronounced significant differences between *word* and *sentence* regarding perceptually affected communication. However, based on our findings, there were no apparent distinctions between *word* and *sentence* except in the emotions of ***Anger*** and ***Sadness***.

Additionally, our study indicated that four emotional states tested at an accuracy level of more than 70%. These findings were compatible with previous research conducted within various linguistic settings (13). Furthermore, language variations suggested that these differences were not impactful to emotional recognition. Reportedly, this identifies beyond conjecture a variety of emotional categories (36, 37). Emotional impacts were significant in *sentence* relating to **sadness**, and absent in differences regarding *word* recognition. According to our findings, support for *sentence* stimuli did not prove advantageous over *word* perception. Most noteworthy was that appropriate emotional perception and vocal speech are inextricably intertwined. Thus, the need for additional theoretical/clinical research deserves additional attention. By applying *sentence* utterances in order to achieve optimal perceptions, this allows the participants to effectively express **sadness** emotion. Conversely, by maximizing the **anger** emotion using *word* utterances appears more pronounced.

Consistent with previous study of Mobes et al. our results regarding [/Anna/] (subject's name), proved instrumental amongst healthy participants (29). For further discussion we will distinguish between findings regarding emotions and speech.

Arousal, defined as a physiological state emphasizing emotional reaction of the subject, reviews the emotions of **anger**, **fear**, **sadness**, **happiness**, and disgust (38, 39). Therefore, we can conclude that physical responses can be a consequence of arousal. Voice parameters can also distinguish varying characteristics of emotions, i.e., fundamental frequency, intensity, duration, and pace (40). Based on this perspective, while fear and anger are expressed differently, panic fear and hot anger in particular are associated with high levels of arousal and audibility (2, 41–44). However, **sadness** is the result of decreased arousal levels coupled with low acoustics.

Expressing short and simple utterances, such as *word*, is preferable to maintaining high arousal levels. Increased vocals therefore produce diminished arousal recognition. The thought behind *sentence* speech, relating to sadness, is clearly defined by low fundamental frequency/intensity, and a longer duration (42). Therefore, **sadness** expression results in longer utterances as evidenced in *sentence* findings.

Previous studies found that speakers often portray stereotypes of emotions and might differ in the quality of their emotional portrayals. For instance, one speaker might be very good at portraying happiness but not fear, whereas another speaker's performance might show the opposite pattern (12, 37). Similarly, past work has shown that emotion categories sharing the same dimension of valence (happiness and surprise) and arousal (anger and fear) are more likely to be conflated (37). Thus, it is plausible that enacted emotions, expressed in isolation, independently with a situational context, belong to the same valence category, which may challenge not only encoders' but also listeners' performance accuracy, thereby leading to ambiguous results. Therefore, our research presented less intertwined results producing a more pristine analysis.

The current study also emphasizes certain limitations: First and foremost, we examine female applicants only, differentiating between various recognitions through multiple speech types. Fast forward to upcoming studies, we anticipate expanding our scope to include gender-related research. Secondly, by expanding our conclusions with cross-cultural language analysis, we can better interpret our research findings. Stimulated speech can present unique challenges and limitations which should be altered in future studies. While the methods of emotional simulation offer high experimental control, the validity of prosodic stimuli derived from these measures is limited (3, 45), thus boosting recognition accuracy (34). Finally, increasing our sample size will produce a more precision based effect.

## Summary And Conclusion

Theoretically, our analysis reveals a novel approach to emotional and vocal effects on our perceptions. With the inputs used in our study, [/Ma/Be/e/met/A/ni/lo/ /Ma/a/mi/na/] and [/Anna/] the word results were found to be more distinct than the anger perception of sentences. Based on this conclusion, we suggest that the connection between specific emotions and type of speech, found in Hebrew language, is differentiated by arousal variations in those affected states. However, to generalize our findings in relation to the types of speech that contribute to emotion recognition, further research is needed. Disorders ranging from nervous system, psychiatric, and neuro-developmental issues are all characterized, amongst others, with diminished expression and perception of emotions. Therefore, it is essential to advance diagnostic assessments, as well as other perceptual/ acoustic measures of voice. Conclusively, the results of this preliminary study, point to the importance of choosing speech tasks, as an integral part of emotional perception.

These findings represent important clinical and research contributions in planning, building, and treating conditions within impaired populations. Additional studies requiring validity testing, should be applied to both healthy and weakened test groups. Similarly, monitoring the sensitivity between multiple types of speech within conditional environments, must be thoroughly examined. Our initial investigation highlights the importance of evaluation and intervention, in order to advance the emotional prosody by voice. This can be applied to many different circumstances including in the field of biometrics identification.

## Data Availability Statement

The raw data supporting the conclusions of this article will be made available by the authors, without undue reservation.

## Ethics Statement

The studies involving human participants were reviewed and approved by IRB Ethics Committees at the Haifa University, Haifa, Israel. The patients/participants provided their written informed consent to participate in this study.

## Author Contributions

R-TS wrote the manuscript and conducted the experiment and data analysis. SS and R-TS designed the study. Both authors have given approval for the current version to be submitted and agree to be accountable for all aspects of the work in ensuring that questions related to the accuracy or integrity of any part of the work are appropriately investigated and resolved.

## Conflict of Interest

The authors declare that the research was conducted in the absence of any commercial or financial relationships that could be construed as a potential conflict of interest.

## Publisher's Note

All claims expressed in this article are solely those of the authors and do not necessarily represent those of their affiliated organizations, or those of the publisher, the editors and the reviewers. Any product that may be evaluated in this article, or claim that may be made by its manufacturer, is not guaranteed or endorsed by the publisher.
